# Influence of the type of acoustic transducer in pure-tone audiometry

**DOI:** 10.1590/2317-1782/20212021019

**Published:** 2022-01-07

**Authors:** Nathália Montandon Born, Mariane da Silva Marciano, Stephanie da Costa Mass, Daniela Polo Camargo da Silva, Renata Coelho Scharlach

**Affiliations:** 1 Curso de Fonoaudiologia, Universidade Federal de Santa Catarina – UFSC - Florianópolis (SC), Brasil.; 2 Departamento de Fonoaudiologia, Universidade Federal de Santa Catarina – UFSC - Florianópolis (SC), Brasil.

**Keywords:** Audiology, Audiometry, Headphones, Transducers, Patient Preference

## Abstract

**Purpose:**

To compare the air-conduction hearing thresholds obtained with different acoustic transducers and verify the users’ preferences regarding them.

**Methods:**

This is a cross-sectional, analytical, observational study with 26 participants aged 18 to 30 years, with normal hearing and no history of exposure to high sound pressure levels or complaints of tinnitus at the time of the assessment. We surveyed their medical history and performed meatoscopy, pure-tone threshold audiometry, speech audiometry, and acoustic immittance. The auditory thresholds were surveyed twice, each time with a different type of acoustic transducer: insert (E-A-RTONE) and circumaural earphones (HDA200). The assessments were performed in a random order, with 5-minute intervals. In the end, we asked the participants which earphones they found more comfortable in the tests. The data were submitted to nonparametric statistical analysis.

**Results:**

Assessing the medians in the auditory threshold survey, the circumaural earphones obtained better results at 250, 500, 2000, and 6000 Hz, while the insert earphones were better at 3000 and 4000 Hz; there were no statistical differences at 1000 and 8000 Hz. The circumaural was elected the most comfortable earphone.

**Conclusion:**

The circumaural earphones had better auditory thresholds at 250, 500, 2000, and 6000 Hz than the insert earphones and were reported by the patients as the most comfortable type of transducer.

## INTRODUCTION

Pure-tone audiometry (PTA), whose objective is to survey the sound intensity threshold at which a person detects sound at various frequencies, is the gold standard examination to assess hearing. It assesses both air-conduction (with earphones) and bone-conduction (with a bone vibrator) thresholds in both ears^(1)^.

Three types of phones are used to obtain air-conduction hearing thresholds for both pure tones and speech stimuli: supra-aural, circumaural, or insert earphones^(2)^.

Supra-aural earphones are the ones most used in audiology clinical practice. It is fitted by pressing the earpad onto the auricle^(3)^, but its disadvantages are the possibility of coupling between the earphone and the ear and the little reliability at low frequencies due to possible air leak^(2)^.

Insert earphones are inserted into the external ear canal with a disposable foam earplug. It ensures minimum contact with the skin and diminishes the area of the head in contact with the sound stimulus, differing from the others^(4)^. Its advantages are the lower risk of collapse and greater interaural attenuation, besides being the earphone with the lowest variability caused by ear leakage, tending to minimize physiological noise in the ear, in contrast with the other types of earphones^(5)^. However, it is subject to variations in the geometry of the external ear canal and difficulties controlling the precise insertion depth^(2)^.

Circumaural earphones, when fitted to the patient, are placed around the auricle. They provide greater comfort because their earpads do not pressure the ear, they considerably reduce the variability in ear leakage and physiological noise in the ear and better attenuates environmental noise^(2,4)^. In general terms, they have greater interaural attenuation than supra-aural earphones, especially at low frequencies^(6)^. Moreover, given their physical characteristics, they are the ones indicated for pure-tone audiometry at high frequencies (9 to 16 kHz)^(7)^. Various studies address the difference in hearing thresholds obtained with acoustic transducers and in different populations. They mostly compare the thresholds obtained with supra-aural and insert earphones – with better results with the insert ones, especially at low frequencies, although the supra-aural earphones sometimes have better results at high frequencies^(3-6,8-12)^. One study has researched the difference between the hearing thresholds obtained with supra-aural and circumaural earphones, showing that the circumaural earphones had better thresholds even at low frequencies^(6)^.

Since the circumaural earphones are used less often in clinical practice, particularly in Brazil, evidently few papers have compared the hearing thresholds obtained with them and the other acoustic transducers. However, audiometers currently enable the use of different types of earphones, which has stimulated their use in clinical practice. This justifies research to provide better knowledge of their performance.

Hence, the objective of this study was to compare the air-conduction hearing thresholds obtained with different acoustic transducers – insert (E-A-RTONE) and circumaural (HDA200) earphones – and verify the users’ preference regarding them.

## METHODS

This is a cross-sectional, analytical, observational study conducted between March and September 2018. We selected for this research people of both genders from a nonprobabilistic convenience sample, employing personal contact and announcement on social media.

This study was analyzed and approved by the Human Research Ethics Committee, under CAAE protocol: 79890817.6.0000.0121, and evaluation report number: 2.537.085. The procedures were explained to all participants, who signed the informed consent form.

The eligibility criteria were as follows: individuals 18 to 30 years old of both genders, without hearing complaints, with hearing thresholds of 25 dBHL or less at 250 to 8000 Hz, speech recognition index (SRI) of at least 88%, and with acoustic reflexes^(13)^.

Subjects with a history of exposure to frequent high occupational and/or recreational sound pressure levels, complaints of tinnitus at the time of the assessment, and any type of obstruction in the external ear canal that might interfere with the assessments were excluded.

All the participants were submitted to the following procedures: medical history survey, meatoscopy, PTA, speech audiometry, and acoustic immittance measures.

In PTA, the air-conduction threshold was surveyed at 250, 500, 1000, 2000, 3000, 4000, 6000, and 8000 Hz, bilaterally, with the descending method. Every time they detected the sound, its intensity was decreased by 10 dB until they stopped responding to the sound. If there was no response, the sound intensity was increased by 5 dB until they detected it again. The hearing threshold was established as the lowest sound intensity they had heard in 50% of the presentations at each frequency tested^(14)^.

To confirm the air-conduction thresholds, we researched the speech recognition threshold (SRT) – i.e., the lowest intensity at which the person recognized at least 50% of the words presented. Then, we surveyed the SRI, presenting in a live voice a list with 25 monosyllables at 40 dBSL, with the three-frequency mean between 500, 1000, and 2000 Hz^(15)^.

We measured the acoustic immittance (tympanometry and acoustic reflex) with an AT235 device (Interacoustics) to exclude the possibility of middle ear changes^(16)^.

The hearing thresholds and SRT were surveyed with two different types of earphones (circumaural and insert) to assess the influence of the type of air-conduction acoustic transducer on the audiological assessment.

The PTA was surveyed with an Astera II audiometer (Otometrics) using circumaural (HDA-200) and insert earphones (E-A-RTONE, manufactured by 3M). The earphones were calibrated according to ISO 389 norms (parts 1, 3, 4, 5, and 8), IEC 60645 (parts 1, 2, and 4), and ISO 8253 (parts 1, 2, and 3).

We defined the order with which each acoustic transducer and ear was assessed in a draw to avoid order effect bias. There were 5-minute intervals in between the collections. Once they were finished, we asked the subjects which earphones they preferred regarding comfort, to classify them as the most and least comfortable ones.

We tabulated the data in an Excel spreadsheet (Microsoft Office Professional Plus, 2013) and submitted them to descriptive and inferential statistical analyses.

Initially, the Kolmogorov-Smirnov test was applied to test the normality of the numerical variables. Since the data did not have a normal distribution (p < 0.0001), we used nonparametric tests. The Mann-Whitney test was used to compare the hearing thresholds between the ears; the Wilcoxon test, to compare the hearing thresholds between the transducers; and the chi-squared test, to analyze the association regarding the comfort in wearing the earphones. The significance level was set at p < 0.05, which is indicated with an asterisk. We used the MedCalc software for data analysis.

## RESULTS

Of the 30 people who agreed to participate in the study, 26 met the eligibility criteria – 16 females and 10 males, aged 18 to 30 years (mean = 23.15 years, median = 23).

Firstly, we compared the hearing thresholds between the ears, for each transducer, and verified no difference between the ears (Mann-Whitney test). Hence, the research data were analyzed considering the total number of ears (n=52).

**Figure 1:**
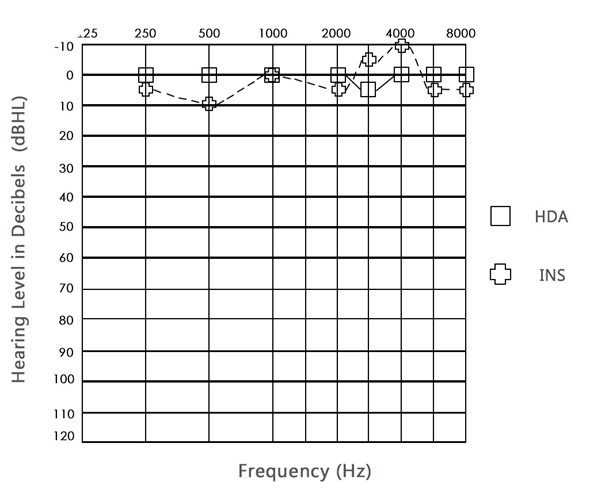
Audiogram comparing the medians of the hearing thresholds for the two transducers per frequency

The median hearing threshold results at 250 to 8000 Hz obtained with the two acoustic transducers – circumaural (HDA-200) and insert (E-A-RTONE) – revealed better results with the circumaural earphone at 250, 500, 2000, 6000, and 8000 Hz, while the result at 1000 Hz was the same for both types of transducers (Figure 1).

Caption: HDA = circumaural earphone; INS = insert earphone

In the comparison between hearing thresholds obtained with each transducer at each frequency tested, the Wilcoxon test revealed better air-conduction hearing thresholds with the circumaural earphone, with a significant difference at all the frequencies, except for 1000 and 8000 Hz (Table 1).

**Table 1:** Descriptive measures of the hearing thresholds (dBHL) comparing the two transducers per frequency (N=52)

Freq (Hz)	Transd	Mean (dBHL)	Median	25^th^ percentile	75^th^ percentile	Minimum	Maximum	P-value
250	HDA	1.34	0	0	5	-10	15	<0.0001*
	INS	7.98	5	5	10	0	25	
500	HDA	- 0.38	0	-5	0	-10	10	<0.0001*
	INS	7.78	10	5	10	-5	15	
1000	HDA	- 0.09	0	-5	5	-10	15	0.340
	INS	0.38	0	-5	5	-10	15	
2000	HDA	1.82	0	-5	5	-10	15	<0.0001*
	INS	6.34	5	2.5	10	-5	20	
3000	HDA	2.40	5	0	5	-5	15	<0.0001*
	INS	- 1.92	-5	-5	5	-10	10	
4000	HDA	1,53	0	-5	5	-10	15	<0.0001*
	INS	- 7.21	-10	-10	-5	-10	5	
6000	HDA	1.15	0	-5	5	-10	20	<0.0001^*^
	INS	5.09	5	0	10	-5	20	
8000	HDA	2.98	0	0	5	-10	25	0.0560
	INS	4.71	5	0	10	-10	20	

Statistical test: Wilcoxon test

* Significant values (p < 0.05)

Caption: Freq = frequency; Transd = transducer; HDA = circumaural earphone; INS = insert earphone

**Figure 2:**
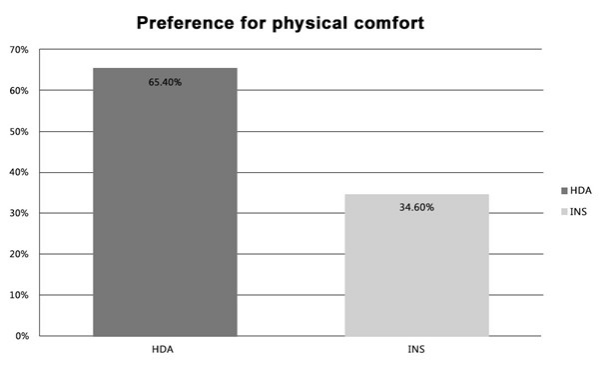
Patients’ preference regarding the physical comfort with each acoustic transducer (N=26)

Regarding the patients’ answers about the physical comfort with the two types of transducers, the chi-squared test showed an association between the type of transducer and their preference – i.e., the HDA transducer was reported by 65.4% of the research participants as the most comfortable one. This preference was statistically significant (p < 0.0001) (Figure 2).

Statistical test: Chi-squared test – p < 0.0001

Caption: HDA = circumaural earphone; INS = insert earphone

## DISCUSSION

Choosing the type of transducer to assess hearing in PTA requires knowledge based on their advantages and disadvantages from both the acoustic and the patient’s perspective. The choice must consider that it is used with different age groups, in different degrees of impairment, aiming to obtain hearing thresholds at different frequencies.

Given the possibility of using supra-aural, insert, or circumaural earphones to obtain air-conduction hearing thresholds at 250 to 8000 Hz, we must compare them and verify the users’ satisfaction with them. We commonly find studies reporting the difference in hearing thresholds obtained with supra-aural and insert earphones in different populations^(5,6,8-12)^, whereas comparisons with circumaural earphones are still scarce^(6,17)^.

A Brazilian study in a young population, whose age range was similar to that in our study (16 to 35 years), without audiological changes, found better hearing thresholds with insert than supra-aural earphones, at 250, 500, 1000, 2000, 3000, and 4000 Hz^(5)^. On the other hand, the present study compared the performance of insert and circumaural earphones and observed that the insert ones were better than the others only at 3000 and 4000 Hz (Table 1). The circumaural, though, was better at 250, 500, 2000, 6000, and 8000 Hz, while there was no difference at 1000 Hz (Table 1). We found no studies in the scientific literature with a similar comparison.

The results show the relevance of choosing circumaural earphones to obtain air-conduction thresholds at 250 to 8000 Hz, as they help improve the values obtained with this examination (Figure 1).

A study conducted in adults showed better thresholds with circumaural^(15)^ than supra-aural earphones. The authors explained it with the fact that circumaural earphones reduce possible air leakage between the earphone and the ear. Hence, they are considerably more reliable at low frequencies, provide greater attenuation of external noise than the supra-aural earphones, diminish the physiological noise in the ear, and are more comfortable^(2,7)^. The greater attenuation of environmental noise provided by the circumaural earphone also qualifies it as the most indicated earphone when the pure-tone audiometry cannot be performed in an acoustically treated setting^(7)^.

Due to the time available to carry out this study, we could not assess whether the differences between the hearing thresholds obtained with the two transducers were actually due to the earphone variable or some variation between test and retest. Nonetheless, a study assessed hearing threshold variations in test and retest with different transducers and verified no difference in hearing thresholds between the assessments, particularly at 500 to 6000 Hz, in comparison with the range from 8000 to 14000 Hz^(18)^. Thus, we believe that the difference in thresholds found in the present research does not result from the test-retest variable, but from the difference between the transducers. The researchers took precautions to avoid variables that might interfere with the result analysis. For instance, we randomized the procedures by drawing lots to define which transducer and ear would be assessed first.

Another rather important aspect in this study was the subjects’ earphone preference regarding comfort in the assessments. The circumaural earphone was elected the most comfortable (Figure 2). This finding can be explained by each earphone’s characteristics. The circumaural earpads are placed around the patient’s auricle, putting less pressure on it, while the insert earphone is fitted into the external ear canal with disposable foam earplugs – which may cause some discomfort^(2)^. A previous study also highlighted the comfort in audiological assessments as an advantage of circumaural earphones^(7)^.

Hence, based on these research findings, we emphasize how important it is for audiologists to know the particularities of the different types of earphones. Thus, they will make better choices not only when providing attention but also when choosing transducers to equip the healthcare service, considering the setting where attention will be provided, the population they will possibly attend, and the type of assessment they will make. Moreover, we believe these research results are relevant to speech-language-hearing clinical practice, as they showed the advantages of using circumaural earphones both for acoustic reasons and the users’ comfort. Some other characteristics of this earphone, already listed in the literature and mentioned in this study, corroborate its use to obtain air-conduction hearing thresholds.

Further research on this topic is warranted, given the scarcity of recent studies comparing the three types of acoustic transducers, particularly in people with unilateral profound hearing loss, to better characterize the interaural attenuation provided by different transducers, especially with circumaural earphones.

## CONCLUSION

The circumaural earphones obtained better hearing thresholds at 250, 500, 2000, and 6000 Hz than the insert ones, besides being the most comfortable transducer as reported by the patients.
