# Supplementary material for: Influence of the type of acoustic transducer in pure-tone audiometry
Source: Codas. 2022 Jan 7;34(3):e20210019. doi: 10.1590/2317-1782/20212021019 (PMC9769422; doi:10.1590/2317-1782/20212021019)
Supplement: Translation

# Translation: A influência do tipo de transdutor acústico na audiometria tonal liminar

**Authors:** Nathália Montandon Born, Mariane da Silva Marciano, Stephanie da Costa Mass, Daniela Polo Camargo da Silva, Renata Coelho Scharlach

**Affiliations:** 1 Curso de Fonoaudiologia, Universidade Federal de Santa Catarina – UFSC - Florianópolis (SC), Brasil.; 2 Departamento de Fonoaudiologia, Universidade Federal de Santa Catarina – UFSC - Florianópolis (SC), Brasil.

**Keywords:** Audiologia, Audiometria, Fones de Ouvido, Transdutores, Preferência do Paciente

## Abstract

**Objetivo:**

Comparar os limiares auditivos por via aérea obtidos em diferentes transdutores acústicos e verificar a preferência do usuário.

**Método:**

Trata-se de um estudo observacional, analítico, transversal, realizado em 26 participantes de 18 a 30 anos, com audição dentro dos padrões de normalidade, sem histórico de exposição a altos níveis de pressão sonora ou queixa de zumbido no momento da avaliação. Realizou-se anamnese, meatoscopia, audiometria tonal liminar, logoaudiometria, e imitanciometria. Os limiares auditivos foram pesquisados duas vezes, cada uma com um tipo de transdutor acústico diferente: fone de inserção (E-A-R Tone) e fone circum-aural (HDA200). A ordem de realização foi aleatória, com intervalos de cinco minutos. Ao final, o participante foi questionado quanto ao conforto dos fones durante os testes. Os dados foram submetidos à análise estatística não paramétrica.

**Resultados:**

Na pesquisa dos limiares auditivos, ao avaliar as medianas, o fone circum-aural apresentou resultados melhores em 250, 500, 2000 e 6000 Hz e o fone de inserção foi melhor em 3000 e 4000 Hz, sem diferença estatística para as frequências de 1000 e 8000 Hz. O fone circum-aural foi eleito o mais confortável.

**Conclusão:**

O fone circum-aural apresentou melhores limiares auditivos em 250, 500, 2000 e 6000Hz quando comparado ao fone de inserção, além de ser o tipo de transdutor mais confortável relatado pelos pacientes.

## INTRODUÇÃO

A Audiometria Tonal Liminar (ATL) é o exame padrão-ouro para avaliar a audição e tem como objetivo pesquisar o limiar de intensidade sonora em que o indivíduo detecta a presença do som em diversas frequências. Por meio desse exame é possível avaliar tanto a audição por via aérea, por meio de fones, quanto por via óssea, por meio de vibrador ósseo, em ambas as orelhas^(1)^.

Para obtenção dos limiares auditivos por via aérea, tanto para tons puros, como para estímulos de fala, é possível utilizar três tipos de fones: supra-aural, circum-aural ou de inserção^(2)^.

O fone supra-aural é o mais utilizado na prática da audiologia clínica, seu ajuste consiste em pressionar o coxim contra o pavilhão auricular^(3)^, como desvantagem há a possibilidade de acoplamento entre o fone de ouvido e a orelha, além disso há confiabilidade reduzida em frequências baixas, devido ao possível vazamento de ar^(2)^.

O fone de inserção fica inserido dentro do meato acústico externo, por meio de plugue de espuma descartável, o que proporciona mínimo contato com a pele e diminui a área da cabeça em contato com o estímulo sonoro, o diferenciando dos demais^(4)^. Suas vantagens consistem na redução do risco de colabamento, aumento da atenuação interaural e também, é o fone que mais reduz a variabilidade causada por vazamentos de ar e tende a minimizar o ruído fisiológico no ouvido em relação aos demais tipos de fone^(5)^. No entanto, está sujeito a variabilidade da geometria do meato acústico externo e dificuldades de controlar a profundidade exata de inserção^(2)^.

O fone circum-aural, quando adaptado no paciente, encontra-se instalado ao redor do pavilhão auricular, o que causa mais conforto ao indivíduo, pois o coxim do fone não gera pressão e reduz consideravelmente a variabilidade do vazamento de ar e o ruído fisiológico no ouvido, além de proporcionar maior atenuação do ruído ambiental^(2,4)^.De forma geral, apresenta atenuação interaural maior em comparação ao fone supra-aural, principalmente em frequências baixas^(6)^. Além disso, por conta de suas características físicas, também é o indicado para a realização da audiometria tonal para as altas frequências (9 a 16kHz)^(7)^. Diversos estudos discutem sobre a diferença dos limiares auditivos obtidos entre os transdutores acústicos em diferentes populações. Em sua maioria, são comparados os limiares obtidos com os fones supra-aural e de inserção, no qual é possível observar que o de inserção obtém melhores resultados, principalmente em frequências baixas, enquanto o supra-aural, algumas vezes, apresenta melhores resultados para frequências altas^(3-6,8-12)^. Já um estudo que pesquisou a diferença dos limiares auditivos obtidos entre os fones supra-aural e circum-aural, foi possível evidenciar que o circum-aural apresentou melhores limiares inclusive para frequências baixas^(6)^.

Como o fone circum-aural é menos utilizado na prática clínica, principalmente na realidade brasileira, fica evidente a escassez de trabalhos que comparam os limiares auditivos obtidos com este fone em relação aos demais transdutores acústicos. Entretanto, atualmente os audiômetros disponibilizam o uso dos diferentes tipos de fones, o que estimulou o seu uso na prática clínica, justificando o melhor conhecimento do seu desempenho por meio de pesquisas.

Desta forma, o objetivo deste estudo foi comparar os limiares auditivos por via aérea obtidos em diferentes transdutores acústicos, de inserção (E-A-RTone) e circum-aural (HDA200), e verificar a preferência do usuário.

## MÉTODO

Tratou-se de um estudo observacional, analítico de delineamento transversal realizado no período de março a setembro de 2018. Para esta pesquisa foram selecionados indivíduos de ambos os sexos, a partir de uma amostra não probabilística por conveniência, por meio de contato pessoal e divulgação da pesquisa em redes sociais.

O presente estudo foi analisado e aprovado pelo Comitê de Ética em Pesquisa com Seres Humanos, sob protocolo CAAE: 79890817.6.0000.0121, parecer número: 2.537.085. Todos os participantes receberam esclarecimentos sobre os procedimentos a serem executados e assinaram o Termo de Consentimento Livre e Esclarecido.

Os critérios de elegibilidade estabelecidos foram: indivíduos de 18 a 30 anos de ambos os sexos, sem queixas auditivas, com limiares auditivos menores ou iguais a 25 dBNA de 250 a 8000 Hz, Índice de Reconhecimento de Fala (IRF) de no mínimo 88% e reflexos acústicos presentes^(13)^.

Foram excluídos os indivíduos com histórico de exposição a níveis de pressão sonora elevados de origem ocupacional e/ou recreacional frequentes, queixa de zumbido no momento da avaliação e algum tipo de obstrução do meato acústico externo que pudesse comprometer as avaliações.

Todos os participantes foram submetidos aos seguintes procedimentos: anamnese, meatoscopia, ATL, logoaudiometria e medidas de imitância acústica.

Na ATL, a pesquisa por via aérea foi realizada nas frequências de 250, 500, 1000, 2000, 3000, 4000, 6000 e 8000 Hz, bilateralmente por meio da técnica descendente. A cada resposta de detecção do som, a intensidade sonora foi reduzida em intervalos de 10 dB até que o indivíduo não respondesse mais ao som. Na ausência de resposta, a intensidade sonora foi elevada com intervalos de 5 dB até que o indivíduo detectasse a resposta. O limiar auditivo correspondeu à menor intensidade sonora ouvida, em 50% das apresentações, para cada frequência testada^(14)^.

Na intenção de confirmar os limiares aéreos, foi pesquisado o Limiar de Reconhecimento de Fala (LRF), ou seja, a menor intensidade na qual o indivíduo reconhece pelo menos 50% das palavras apresentadas. Em seguida, foi realizada a pesquisa do índice de reconhecimento de fala, no qual foi apresentada uma lista de 25 palavras monossílabas a 40 dBNS, da média tritonal de 500, 1000 e 2000 Hz, à viva voz^(15)^.

Para se excluir qualquer alteração de orelha média, foram realizadas as medidas de imitância acústica (timpanometria e a pesquisa do reflexo acústico), por meio do equipamento AT235 (Interacoustics)^(16)^.

Para avaliar a influência do tipo de transdutor acústico de via aérea na avaliação audiológica, os limiares auditivos e o LRF foram realizados com dois diferentes tipos de fones, o circum-aural e o de inserção.

A ATL foi realizada por meio do audiômetro Astera II (Otometrics) com uso do fone circum-aural (HDA-200) e com o fone de inserção (modelo E-A-R TONE da 3M). Os fones foram calibrados de acordo com as Normas ISO 389 Partes 1, 3, 4, 5 e 8 IEC 60645 partes 1, 2, 4 e ISSO 8253 Partes 1, 2, 3.

A ordem de avaliação para cada transdutor acústico e orelha foram feitos a partir de um sorteio, no intuito de não se ter prejuízo devido ao efeito de ordem e foram realizados intervalos de cinco minutos entre as coletas. Ao término os indivíduos foram questionados quanto à preferência em relação ao conforto, podendo classificar como o mais confortável e o menos confortável.

Os dados foram tabulados em uma planilha do Excel (Microsoft Office Professional Plus, 2013) e submetidos à análise estatística descritiva e inferencial.

Inicialmente aplicou-se o teste Kolmogorov-Smirnov para testar a normalidade das variáveis numéricas. Uma vez que os dados não mostraram distribuição normal (p < 0,0001), foram utilizados testes não paramétricos. O teste de Mann-Whitney foi utilizado para a comparação dos limiares auditivos entre as orelhas, o Teste de Wilcoxon na comparação dos limiares auditivos entre os transdutores e o Teste de Qui Quadrado nas análises de associação quanto ao conforto do uso dos fones. Foi estabelecido como nível de significância quando p < 0,05, sendo estes marcados com um asterisco. O *software* MedCalc foi utilizado para a análise dos dados.

## RESULTADOS

Dos 30 indivíduos que aceitaram participar do estudo, 26 atenderam os critérios de elegibilidade, sendo 16 do sexo feminino e dez do sexo masculino, com idade entre 18 e 30 anos (média = 23,15 anos, mediana = 23).

Inicialmente, os limiares auditivos foram comparados entre as orelhas, para cada transdutor e verificou-se que não houve diferença entre as orelhas (Teste de Mann-Whitney). Sendo assim, os dados desta pesquisa foram analisados considerando o número total de orelhas (n=52).

Os resultados dos limiares auditivos medianos de 250 a 8000Hz obtidos com os diferentes transdutores acústicos, circum-aural (HDA-200) e de inserção (E-A-R TONE) demonstraram que o fone circum-aural apresentou melhores resultados para as frequências de 250, 500, 2000, 6000 e 8000 Hz, sendo que 1000 Hz permaneceu igual para os dois tipos de transdutores (Figura 1).

**Figura 1:** Audiograma comparando as medianas dos limiares de audibilidade, para os dois transdutores por frequência

Legenda: HDA = fone circum-aural; INS = fone de inserção

Na comparação dos limiares audiométricos obtidos para cada transdutor em cada frequência sonora testada, o teste de Wilcoxon evidenciou melhores limiares auditivos por via aérea para o fone circum-aural, com diferença significativa em todas as frequências testadas, com exceção de 1000 e 8000Hz (Tabela 1).

**Tabela 1:** Medidas descritivas dos limiares de audibilidade (dBNA), comparando os dois transdutores por frequência (N=52)

Freq (Hz)	Transd	Média (dBNA)	Mediana	25º percentil	75º percentil	Mínimo	Máximo	Valor de P
250	HDA	1,34	0	0	5	-10	15	<0,0001*
	INS	7,98	5	5	10	0	25	
500	HDA	- 0,38	0	-5	0	-10	10	<0,0001*
	INS	7,78	10	5	10	-5	15	
1000	HDA	- 0,09	0	-5	5	-10	15	0,340
	INS	0,38	0	-5	5	-10	15	
2000	HDA	1,82	0	-5	5	-10	15	<0,0001*
	INS	6,34	5	2,5	10	-5	20	
3000	HDA	2,40	5	0	5	-5	15	<0,0001*
	INS	- 1,92	-5	-5	5	-10	10	
4000	HDA	1,53	0	-5	5	-10	15	<0,0001*
	INS	- 7,21	-10	-10	-5	-10	5	
6000	HDA	1,15	0	-5	5	-10	20	<0,0001*
	INS	5,09	5	0	10	-5	20	
8000	HDA	2,98	0	0	5	-10	25	0,0560
	INS	4,71	5	0	10	-10	20	

Teste estatístico: Teste Wilcoxon

* Valores significativos (p < 0,05)

Legenda: Freq = frequência; Transd = transdutor; HDA = fone circum-aural; INS = fone de inserção

Quanto às respostas ao conforto físico de cada paciente em relação aos diferentes tipos de transdutores, o teste Qui quadrado mostrou associação entre o tipo de transdutor e a preferência, ou seja, o transdutor HDA foi relatado por 65,4% dos participantes da pesquisa como sendo o mais confortável, sendo esta preferência estatisticamente significante (p < 0,0001) (Figura 2).

**Figura 2:** Preferência dos pacientes quanto ao conforto físico de cada transdutor acústico (N=26)

Teste estatístico: Teste Qui Quadrado – p < 0,0001

Legenda: HDA = fone circum-aural; INS = fone de inserção

## DISCUSSÃO

Na ATL, a escolha do tipo de transdutor na avaliação da audição do paciente requer o conhecimento baseado nas vantagens e desvantagens tanto do ponto de vista acústico como do próprio paciente, já que é utilizado em diferentes faixas etárias, em diferentes graus de comprometimento e para a obtenção dos limiares auditivos em diferentes frequências.

Diante da possibilidade da utilização dos fones supra-aural, de inserção e circum-aural, na obtenção dos limiares auditivos por via aérea na faixa de 250 a 8000Hz, a comparação entre eles, bem como a satisfação do usuário se fazem necessárias. Comumente encontramos estudos que relatam a diferença dos limiares auditivos obtidos entre os fones supra-aural e de inserção em diferentes populações^(5,6,8-12)^, enquanto que a comparação com o fone circum-aural ainda é escassa^(6,17)^.

Estudo brasileiro em população jovem, com faixa etária semelhante ao presente estudo (16 a 35 anos), sem alterações audiológicas, encontrou limiares auditivos obtidos com fone de inserção melhores quando comparados com os obtidos com o fone supra-aural, nas frequências de 250, 500, 1000, 2000, 3000 e 4000 Hz^(5)^. Por outro lado, no presente estudo, ao comparar o desempenho do fone de inserção com o fone circum-aural observou-se que o primeiro (fone de inserção) foi melhor que o circum-aural apenas para as frequências de 3000 e 4000 Hz (Tabela 1). Já o circum-aural foi melhor em 250, 500, 2000, 6000 e 8000Hz, sem diferença para 1000Hz (Tabela 1). Na literatura científica, não foram encontrados estudos que realizaram tal comparação.

Os resultados mostram a relevância da escolha do fone circum-aural na obtenção dos limiares de via aérea de 250 a 8000Hz, já que auxilia na melhora dos valores na obtenção de limiares aéreos (Figura 1).

Um estudo realizado com indivíduos adultos mostrou melhores limiares para o fone circum-aural^(15)^ em comparação ao fone supra-aural e a explicação dada pelos autores se deve ao fato do fone circum-aural reduzir o possível vazamento de ar entre o fone e a orelha, sendo consideravelmente mais confiável em frequências baixas, além de oferecer uma maior atenuação do ruído externo quando comparado ao fone supra-aural, diminuir o ruído fisiológico no ouvido do indivíduo e ser mais confortável^(2,7)^. A maior atenuação do ruído ambiental proporcionada pelo fone circum-aural o elege também como fone mais indicado em situações nas quais a audiometria tonal não pode ser realizada em um ambiente acusticamente tratado^(7)^.

Em virtude do tempo necessário para a realização do presente estudo, não foi possível avaliar se as diferenças dos limiares auditivos, obtidas entre os transdutores, foram realmente pela variável “fone de ouvido” ou por uma variação entre teste e reteste. Entretanto, em um estudo realizado com o intuito de avaliar a variação dos limiares auditivos em situações de teste e reteste para diferentes transdutores, verificou-se que não houve diferença dos limiares auditivos entre as avaliações, principalmente na faixa de frequência de 500 a 6000Hz quando comparado à faixa de 8000 a 14000Hz^(18)^. Desta forma, acredita-se que a diferença de limiares encontrada na presente pesquisa não é decorrente da variável teste e reteste e sim da diferença entre os transdutores. Com o intuito de evitar variáveis que pudessem interferir na análise dos resultados, os pesquisadores tomaram alguns cuidados como, por exemplo, aleatorizar os procedimentos, realizando-se sorteio para definir qual seriam o transdutor e a orelha na primeira avaliação.

Outro aspecto muito importante, realizado neste estudo, foi a preferência dos indivíduos em relação ao conforto de cada fone, durante as avaliações. O fone circum-aural foi eleito o mais confortável (Figura 2). Este achado pode ser explicado pelas características de cada fone, visto que o fone circum-aural quando colocado no paciente o coxim do fone encontra-se ao redor do pavilhão auricular, o que gera uma menor pressão sobre o pavilhão, já o fone de inserção fica adaptado dentro do meato acústico externo por meio de plugues de espuma descartáveis, o que pode gerar certo incômodo^(2)^. Estudo anterior também ressaltou como vantagem do fone circum-aural o seu conforto durante a avaliação audiológica^(7)^.

Desta forma, a partir dos achados desta pesquisa ressalta-se a importância do conhecimento do audiologista sobre as particularidades dos diferentes tipos de fones para se ter uma melhor escolha, seja no momento dos seus atendimentos, como no momento de escolha do transdutor para equipar seu serviço, pensando no local em que eles serão realizados, a população que possivelmente atenderá e o tipo de avaliação que realizará. Ademais, acredita-se que os resultados desta pesquisa são de relevância para a clínica fonoaudiológica, pois mostrou as vantagens do uso fone circum-aural tanto para os aspectos acústicos como para o conforto do usuário. Além disso, outras características deste fone, já elencadas na literatura e citadas neste estudo, corroboram para que este seja um fone utilizado para a obtenção dos limiares auditivos por via aérea.

Sugerem-se pesquisas com este tema, visto a escassez de estudos recentes e que comparem os três tipos de transdutores acústicos, principalmente em indivíduos com perda auditiva profunda unilateral, a fim de caracterizar melhor a atenuação interaural gerada por diferentes transdutores, especialmente com o fone circum-aural.

## CONCLUSÃO

O fone circum-aural apresentou melhores limiares auditivos em 250, 500, 2000, 6000Hz quando comparado ao fone de inserção, além de ser o tipo de transdutor mais confortável relatado pelos pacientes.
